# Sexual Impotence in the Male and Female

**Published:** 1888-04

**Authors:** 


SEXUAL IMPOTENCE IN THE MALE AND FEMALE. By William A. Hammond, M. D., Professor of Diseases of the Nervous System in the New York Post-Graduate Medical School. Detroit: George S. Davis.

Like the work of Dr. Gross this book does not now require extended criticism. In this edition Dr. Hammond has added four chapters on sexual impotence in the female. Dr. Hammond is intellectually the peer of any physician in America, and can, therefore, make a book very interesting; but the pleasure experienced in reading this book is very like the fascination which causes people to read Zola's works, and it may well be doubted whether any good has resulted from its publication.

